# Speech-in-noise representation in the aging midbrain and cortex: Effects of hearing loss

**DOI:** 10.1371/journal.pone.0213899

**Published:** 2019-03-13

**Authors:** Alessandro Presacco, Jonathan Z. Simon, Samira Anderson

**Affiliations:** 1 Department of Otolaryngology, University of California, Irvine, CA, United States of America; 2 Center for Hearing Research, University of California, Irvine, CA, United States of America; 3 Department of Electrical & Computer Engineering, University of Maryland, College Park, MD, United States of America; 4 Department of Biology, University of Maryland, College Park, MD, United States of America; 5 Institute for Systems Research, University of Maryland, College Park, MD, United States of America; 6 Neuroscience and Cognitive Science Program, University of Maryland, College Park, MD, United States of America; 7 Department of Hearing and Speech Sciences, University of Maryland, College Park, MD, United States of America; Australian Research Council Centre of Excellence in Cognition and its Disorders, AUSTRALIA

## Abstract

Age-related deficits in speech-in-noise understanding pose a significant problem for older adults. Despite the vast number of studies conducted to investigate the neural mechanisms responsible for these communication difficulties, the role of central auditory deficits, beyond peripheral hearing loss, remains unclear. The current study builds upon our previous work that investigated the effect of aging on normal-hearing individuals and aims to estimate the effect of peripheral hearing loss on the representation of speech in noise in two critical regions of the aging auditory pathway: the midbrain and cortex. Data from 14 hearing-impaired older adults were added to a previously published dataset of 17 normal-hearing younger adults and 15 normal-hearing older adults. The midbrain response, measured by the frequency-following response (FFR), and the cortical response, measured with the magnetoencephalography (MEG) response, were recorded from subjects listening to speech in quiet and noise conditions at four signal-to-noise ratios (SNRs): +3, 0, -3, and -6 dB sound pressure level (SPL). Both groups of older listeners showed weaker midbrain response amplitudes and overrepresentation of cortical responses compared to younger listeners. No significant differences were found between the two older groups when the midbrain and cortical measurements were analyzed independently. However, significant differences between the older groups were found when investigating the midbrain-cortex relationships; that is, only hearing-impaired older adults showed significant correlations between midbrain and cortical measurements, suggesting that hearing loss may alter reciprocal connections between lower and higher levels of the auditory pathway. The overall paucity of differences in midbrain or cortical responses between the two older groups suggests that age-related temporal processing deficits may contribute to older adults’ communication difficulties beyond what might be predicted from peripheral hearing loss alone; however, hearing loss does seem to alter the connectivity between midbrain and cortex. These results may have important ramifications for the field of audiology, as it indicates that algorithms in clinical devices, such as hearing aids, should consider age-related temporal processing deficits to maximize user benefit.

## Introduction

Speech understanding significantly degrades with aging, particularly in noisy environments. Older adults show some ability to cope with their communication problems in quiet, but largely fail to do so in noise, where the need to segregate two or more speech streams is compromised [1]. Behavioral studies have suggested that deficient auditory temporal processing is a key factor in explaining age-related difficulties with understanding speech in noise [2–8]. Several electrophysiological studies have suggested that these communication problems are linked to temporal processing deficits arising from subcortical [9–19] and cortical regions [18–30].

Two of the main biomarkers used to investigate aging deficits are the response strength, represented by the amplitude, and the response latency, which is represented by timing of the main peaks measured in the averaged time domain neural response. Several aging studies investigating both midbrain and cortical activity in the same subject have revealed that the amplitude response in these two regions of the auditory pathway may be altered in different ways [18, 19, 31]. Specifically, aging is associated with a decline in phase-locked activity in the inferior colliculus, likely caused by a local imbalance between excitatory and inhibitory mechanisms, which disrupts the precise coding needed to represent a complex stimulus at brainstem/midbrain levels [11]. Temporal jitter or decreased afferent input associated with a reduction in auditory nerve fibers [32, 33] may also be responsible for the significant drop in phase locking or amplitude observed in the midbrain frequency-following response (FFR).

In contrast to the midbrain amplitude declines, cortical responses are augmented in older compared to younger adults [18–22]. Several differing mechanisms may contribute to the exaggerated cortical responses observed in older adults. A central compensatory mechanism may be triggered to cope with decreased or degraded input from the brainstem/midbrain, in line with recent results [34] that demonstrate restoration of auditory object representation at the cortical level even when auditory neuropathy abolishes the auditory brain stem response. The augmented cortical response may also result from decreased cortical connectivity among the areas of the brain involved in speech comprehension, such as inferior and middle frontal gyrus, and subsequent increases in redundant local processing [35]. This loss of connectivity may cause reduced coherence among different areas of the brain involved in speech comprehension, such as inferior and middle frontal gyrus, along with an unusual activation of larger areas of the brain, including the cingulo-opercular network and the dorsal prefrontal cortex [36, 37]. It is also possible that the exaggerated cortical response arises from age-related alteration in inhibitory neural mechanisms in the cortex that lead to an increase in spontaneous and evoked neural firing rates [26–29].

While the findings of these aging studies are quite compelling, they sidestep issues arising from concomitant peripheral hearing loss that often accompany aging and compromise speech understanding [38, 39]. Peripheral deficits may play an important role in altering the final representation of the auditory object, as a progressive loss of cochlear synapses and nerve fibers has been observed in aging animal models [33, 40]. A number of studies have shown that decreased audibility affects auditory temporal processing [17, 30, 38, 39, 41–45]. Peripheral hearing loss may lead to reorganization of cortical activity and changes in cortical resource allocation [46–49], so that additional brain resources (that would otherwise be allocated for other sensory or cognitive functions) would need to be harnessed to assist with auditory tasks. Alteration of local inhibitory control in different parts of the auditory system, as a direct consequence of hearing loss, has also been reported in several animal studies [50–55]. A few studies have also demonstrated differing hearing loss effects for midbrain vs. cortical processing, similar to differing aging effects in these regions. For example, greater hearing loss is associated with reduced fMRI activation in subcortical areas without concomitant decreases in cortical activation [56]. Furthermore, greater degrees of hearing loss are related to decreased FFR pitch representation but increased cortical magnitudes [31]. In some of these studies, though, hearing loss and aging overlap, making it difficult to disentangle the separate contributions of each to the problems experienced by older adults in noisy environments. It is plausible that degraded auditory temporal processing associated with age-related peripheral hearing loss may explain why hearing aids often fail to improve speech understanding in noise, notwithstanding the boost in audibility. Despite advances in hearing aid technology, algorithms that focus on both increasing audibility within the auditory dynamic range of the listener and on improving the speech signal-to-noise ratio (SNR) may still not restore temporal precision degraded by aging.

Results from our previous studies [18, 19] conducted on normal-hearing older adults showed a substantial degradation (e.g. significantly lower amplitude) of the midbrain response and an exaggeration (or overrepresentation) of the cortical response, both in quiet and noise, with respect to normal-hearing younger adults. Overall, those results suggested that temporal processing deficits in the central auditory system contribute to speech-in-noise problems experienced by older adults. Additionally, since evidence suggests that attention and inhibitory control significantly affect the ability to segregate speech streams [57, 58], the relationships among these cognitive variables and the overrepresentation of the neural response were also investigated [18]. This relationship could indeed be important, as several studies [59–61] have suggested that additional cognitive demands imposed by degraded stimuli have the potential to force older adults to involuntarily allocate more resources (e.g. attention), which would otherwise be available for secondary tasks, in order to encode the target auditory stimulus. These findings are of critical importance, as a number of experiments [57, 58] have shown that individuals who perform well on cognitive tasks that measure attention and inhibition tend to be less distracted by competing talkers and to be more successful in focusing on the target auditory stimulus, even if measured with a purely visual task such as the Flanker task [62].

Interestingly, these same earlier studies demonstrated that the cortical response, as measured by stimulus reconstruction accuracy, is negatively correlated with cognitive scores, but only in older adults. This finding reinforces the theory that an exaggerated cortical response is a biomarker potentially representing a failure in efficiently encoding auditory information, so that an increase in neural resources is allocated to the task in individuals with poorer cognitive function. In fact, as reported in our previous study [18], this negative correlation would be in agreement with the hypothesis that higher reconstruction accuracy may not beneficial for older adults, but may be the result of an abnormal increase in neural current, possibly caused by an imbalance between excitatory and inhibitory mechanisms. Peripheral hearing loss, however, could not be ruled out as a contributing factor to these findings; despite having normal to borderline-normal audiometric thresholds, the older adults in these previous studies did have significantly worse hearing thresholds than the younger adults at most of the frequencies tested. It would be important to determine the role of peripheral hearing loss in the cognitive-neural relationship, as several behavioral studies [63–65] have suggested that decline in hearing sensitivity has the potential to accelerate cognitive decline.

To investigate the role of peripheral hearing loss on auditory temporal processing and cognitive-neural relationships, data collected from previous studies [18, 19] was compared with new data collected from a sample of older hearing-impaired (OHI) adults. Electroencephalography (EEG) was used to record FFRs thought to originate primarily from the midbrain, since this neuroimaging technique is sensitive to subcortical activity [66, 67] and has temporal resolution of the order of milliseconds. The strength of the FFR response was used to measure the ability of midbrain neurons to synchronize in response to the stimulus, the ability of the midbrain neurons to fire similarly in quiet and in noise was assessed by the quiet-to-noise correlation, and the ability of the brain to follow the auditory input was quantified by calculating the correlation between stimulus and neural response.

Magnetoencephalography (MEG) is a neuroimaging technique that records magnetic signals primarily generated by electrical currents flowing between neurons [68]. To obtain a measure of the fidelity of the neural representation of that speech envelope, MEG was used to quantify the reconstruction of the low-frequency speech envelope from cortical activity. MEG activity was analyzed in three integration windows (150, 300, and 500 ms) to determine if older adults require a longer processing time than younger adults to maximize reconstruction of the speech envelope.

Because magnetic fields are not affected by volume conduction and thus are not smeared by the scalp, skull and other biological tissues, MEG can be used to effectively record low frequency neural oscillations originating in the cortex. MEG is not considered appropriate for midbrain analysis because of its relative insensitivity to subcortical activity [69]. This observation is supported by recent results [66] suggesting that the brainstem contribution to FFRs may be three times as large as the contribution of the auditory nerve and auditory cortex. Observations of cortical generators in MEG FFR studies [70] may therefore be possible only due to the insensitivity of MEG to activity generated by sources located in deep brain regions [66, 68].

Our main hypothesis is that while the presence of hearing loss exacerbates speech understanding difficulties (due to reduced audibility and decreased frequency selectively), it does not significantly increase the age-related temporal processing deficits observed in normal-hearing older adults (ONH). This hypothesis stems from previous studies that demonstrated effects of aging, but not hearing loss, on temporal processing. Studies that include younger and older adults, both with normal hearing and with hearing loss, are rare due to the difficulty in recruiting young people with hearing loss. However, one pair of studies did successfully recruit these four groups and assessed their perception of stimulus duration and stimulus temporal order, two tasks that rely on the central auditory system [71, 72]. They found that aging, rather than hearing loss, affected performance on these tasks. Using a binaural-masking-level-difference paradigm, results from a recent study [73] also demonstrated that aging, but not hearing loss, was associated with a decrease in the processing of binaural temporal fine structure cues in both behavioral and cortical EEG assessments.

In the previous studies on which this work builds, neural speech processing was measured in quiet and at SNRs ranging from -6 to +3 dB [18, 19]. The use of multiple SNRs enabled measuring the extent to which progressive levels of noise degrade neural responses. An additional motivation to use multiple SNRs stems from previous studies that demonstrated age-group differences in noise-related reductions in amplitude at only higher levels of masking. Recent results [74] found that broadband noise as low as 20 to 30 dB of effective masking reduced auditory brainstem response amplitudes in younger adults, but 70 dB of effective masking was needed to reduce amplitudes in older adults. The age-group differences in masking effects may arise from different baseline levels of neural synchrony between younger and older adults associated with loss of auditory nerve fibers or cochlear synaptopathy [33, 40]. Hearing loss may also reduce sensitivity to changes in SNR; recent results [45] showed that neural tracking of the attended speech signal, quantified by cross-correlating the EEG signal with the speech envelope, improved with increases in SNR in participants with normal hearing, but did not improve in participants with hearing loss. Therefore, the use of multiple SNRs permits us a more comprehensive assessment of aging and hearing loss effects on auditory temporal processing.

In this study, we hypothesize that the midbrain activity will be equally degraded (e.g. reduced amplitude) in ONH and OHI adults relative to younger normal-hearing (YNH) adults. Similarly, in the cortex we hypothesize the reconstruction fidelity, or the ability of the brain to track the speech envelope, to be equally overrepresented in both ONH and OHI adults relative to YNH adults. Finally, based on the results from the previous study that suggested that the overrepresentation of the cortical response is a negative rather than positive effect of aging [18], we expect to find a negative correlation between cognitive performance and cortical responses in both groups of older adults. YNH listeners were included in this analysis because they represent a gold standard of auditory neural processing, against which to investigate similarities and dissimilarities between the two groups of older listeners.

## Materials and methods

### Participants

The new set of participants comprised 14 OHI adults (62–86 years old, mean ± sd 71.28 ± 6.26, 9 males) recruited from the Maryland, Washington D.C. and Virginia areas. The data from these subjects are compared to the data obtained from the 17 YNH adults (18–27 years, mean ± sd 22.23 ± 2.27, 3 male) and 15 ONH adults (61–73 years old, mean ± sd 65.06 ± 3.30, 5 males) in our previous studies [18, 19]. The OHI group was significantly older than the ONH group (*p* = 0.002). All procedures were reviewed and approved by the Institutional Review Board (IRB) of the University of Maryland. Participants gave written informed consent and were paid for their time.

**Fig 1:**
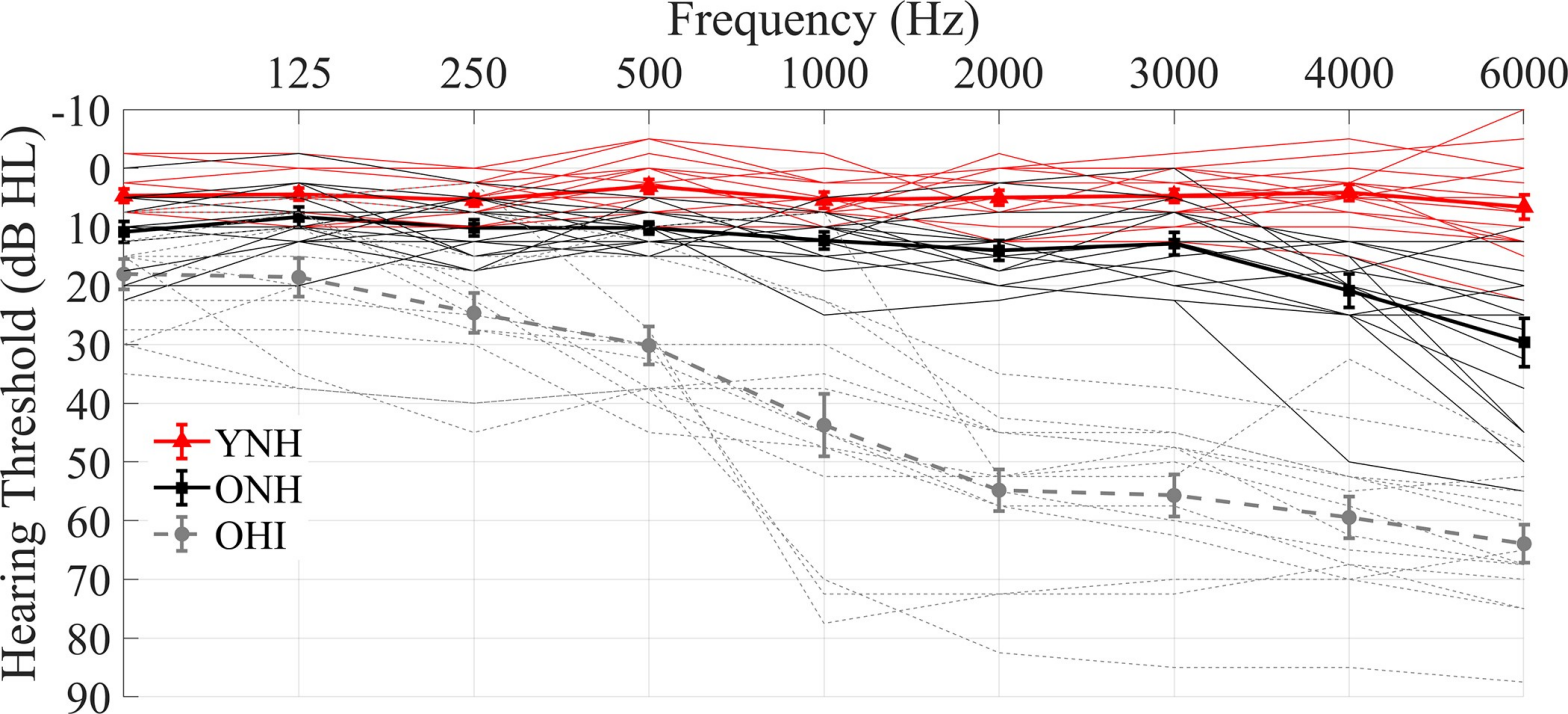
**Audiogram (mean ± 1 SE) of the averages (right and left) of YNH (red line), ONH (black line) and OHI (dashed-gray line) adults.** Thin lines represent the PTA of the individual subjects, while thick lines represent the grand average. YNH and ONH have pure-tone averages ≤ 25 dB HL from 125 to 4000 Hz, while OHI have an average hearing loss across 500–4000 Hz of 26 dB HL or worse.

YNH and ONH adults had normal to borderline-normal audiometric thresholds (air conduction thresholds ≤ 25 dB HL from 125 to 4000 Hz bilaterally). OHI adults had sensorineural hearing loss with average pure-tone thresholds from 500–4000 Hz ≥ 26 dB HL with no thresholds in this frequency range > 90 dB HL. In the three groups, there was no interaural asymmetry (> 15 dB HL difference at no more than two adjacent frequencies) and no air-bone gaps greater than 10 dB were noted at any frequency. Fig 1 shows average audiograms for the three groups. All participants from the three groups had normal IQ scores [≥ 85 on the Wechsler Abbreviated Scale of Intelligence [75]] and were not significantly different on IQ (F_[2,43]_ = 0.429, *p* = 0.654). ONH and OHI adults were also not significantly different in sex (Fisher’s exact, *p* = 0.143). Both groups of older adults were screened for dementia on the Montreal Cognitive Assessment (MoCA) [76]. The mean ± sd of the dementia screening was 26.9 ± 2.7 for ONH and 26.7 ± 2 for OHI adults and no significant differences were found between the two older listener groups (F_[__1__,__27__]_ = 0.027, *p* = 0.871). All participants included in this study scored at or above the screening criterion of 22. The Edinburgh Handedness Inventory was also administered to our participants to assess their right or left hand dominance. All the participants, but two YNH and one ONH, were right-handed. Because of the established effects of musicianship on subcortical auditory processing [77, 78], professional musicians were excluded. All participants participated in both the EEG and MEG study and spoke English as their first language. EEG and MEG data for each participant were collected in two separate sessions.

### Speech intelligibility

The Quick Speech-in-Noise test (QuickSIN) [79] was used to quantify the listener’s ability to recognize sentences presented in four-talker babble. Speech was presented at 70 dB HL to all participants, except for two of the participants with hearing loss who required 80 dB HL to ensure adequate audibility to perform the task, using procedures as suggested in the QuickSIN procedure manual.

### EEG: Stimuli and recording

A 170-ms /da/ [10] was synthesized at a 20 kHz sampling rate with a Klatt-based synthesizer [80]. The stimulus was presented at 75 peak dB SPL diotically with alternating polarities at a rate of 4 Hz through electromagnetically shielded insert earphones (ER-1; Etymotic Research) via Xonar Essence One (ASUS) sound card using Presentation (Neurobehavioral Systems, Inc.). FFRs were recorded in quiet and in the presence of a single female competing English speaker narrating *A Christmas Carol* by Charles Dickens (https://librivox.org/a-christmas-carol-version-6-by-charles-dickens) presented at 4 noise levels: +3, 0, -3, and -6 dB SNRs as described in [19]. The EEG data were recorded at a sampling frequency of 16384 Hz using the Biosemi ActiABR-200 acquisition system (BioSemi B.V.) using the same montage and filter specifications as described in [19]. During the recording session (~2 hr), participants sat in a recliner and watched a silent, captioned movie of their choice to facilitate a relaxed yet wakeful state. A minimum 2300 sweeps were collected for each subject for each of the five total noise levels tested.

### EEG: Data analysis

EEG data were analyzed in MATLAB (MathWorks, version R2011b) after being converted into MATLAB format with the function pop_biosig from EEGLab [81]. The offline analysis of the data was the same as the one described in our previous study [19]. Briefly, the first 1000 sweeps per polarity (a total of 2000 per subject and per condition) with amplitude in the ±30 μV range were first band-pass filtered from 70 to 2000 Hz and then averaged to create a final average used for the statistical analyses. In 2 conditions (-3 and -6 dB) and only for subject S06 with hearing impairment, 1512 and 1938 sweeps, respectively, were used for the analysis, because the remaining sweeps recorded did not pass the artifact rejection criterion. Sweeps of both polarities were added to minimize the influence of cochlear microphonic and stimulus artifact on the response and to maximize the envelope response [82–84]. Three different analyses were performed on the EEG data: 1) the root-mean square (RMS) value of the response was used to measure the strength of the response, 2) the quiet-to-noise response analysis, which consisted of taking the cross-correlation between the responses in quiet and in noise at lag zero, was used to measure the consistency between the responses in quiet and noise and 3) the stimulus-to-response correlation, which consisted of first extracting the envelope of the stimulus by using the absolute value of the analytic signal of the stimulus, then band-pass filtering the envelope using the same filter as for the response (70–2000 Hz) and then correlating the filtered envelope with the average response. This last analysis was used to analyze the ability of the brain to effectively follow the stimulus envelope of the stimulus. The three above-mentioned analyses were applied to the transition (18–68 ms) and steady-state (68–170 ms) regions. The transition region reflects the syllable transition from the consonant /d/ to the vowel /a/, whereas the steady-state region represents the unchanging vowel region. The transition region was chosen to investigate older adults’ ability to cope with rapid changes in the frequency components, while the steady-state region was chosen to study the response to the periodicity of the stimulus [10].

### MEG recording

The task and stimuli were the same as the ones described in our previous study [19]. Briefly, the stimuli for the target male speaker (foreground) were extracted from the book *The Legend of Sleepy Hollow* by Washington Irving (https://librivox.org/the-legend-of-sleepy-hollow-by-washington-irving), whereas the competing female English speaker was the same used in the EEG experiment. Five different conditions were played: quiet, +3, 0, -3 and -6 dB SNR. The same segment was played for quiet and -6 dB; the quiet condition was always played last. In order to ensure a sufficient level of attention by the subject on the foreground segment, participants were asked beforehand to count the number of times a specific word or name was uttered in the story. The level delivered to normal-hearing participants was approximately 70 dB SPL when presented with a solo speaker (i.e. audio stimulus played without the presence of the female talker) to the participants’ ears with a 50 Ω transducer and sound tubing (E-A-RTONE 3A; Etymotic Research), attached to E-A-RLINK foam plugs inserted into the ear canal. Two participants who reported that they could not understand the story line clearly at 70 dB SPL in quiet required increasing the level of the noise-free speech up to 75 dB SPL (in 1-dB steps) until they could hear it clearly. Though this procedure is not optimal, it is unlikely that this minor change in intensity level had a significant effect on the results since the reconstruction of the speech envelope is insensitive to the loudness of the target speaker [85]. The entire acoustic delivery system was equalized to give an approximately flat transfer function from 40 to 3000 Hz, thereby encompassing the range of the presented stimuli. Neuromagnetic signals were recorded using a 157-sensor whole head MEG system (Kanazawa Institute of Technology, Kanazawa, Japan) in a magnetically shielded room as described in [85].

### MEG: Data analysis

The same data analysis described in our previous study [19] was applied to the data. Briefly, the data collected from the 157 sensors were processed by the denoising source separation (DSS) algorithm [86, 87] and the first 6 DSS components were band-passed from 1 to 8 Hz and then used to reconstruct the speech envelope by means of a linear reconstruction matrix estimated via the Boosting algorithm [85, 88]. The speech envelope was extracted by taking the magnitude of the analytic signal of the stimulus and then band-passing from 1 to 8 Hz. Data were analyzed by using 3 different integration windows: 500, 350 and 150 ms. As described in our previous study [19], these values refer to the time shift imposed on the data with respect to the onset of the speech and to the corresponding integration window of the reconstruction matrix. As conjectured in our previous study [19], if processing time for younger and older adults is the same, then their performance should follow the same pattern as the integration window changes. Conversely, if older adults require more time to process the information because of the possible presence of temporal processing deficits, then the narrowing of the integration window should negatively affect their performance more than for younger adults. The noise floor was calculated by using the neural response recorded from each noise level tested to reconstruct the speech envelope of a stimulus that was not played during any of the conditions tested (a 1-minute speech segment extracted from a different story) to allow the noise floor to incorporate contributions from potential overfitting.

### Cognitive test

The Flanker Inhibitory Control and Attention Test of the National Institutes of Health Cognition Toolbox was used to measure executive function (ability to inhibit visual attention to irrelevant tasks) and attention. Participants were shown a series of arrows and were asked to determine as quickly as possible the direction of the middle arrow by pressing either the left or right arrow on the keyboard. The unadjusted scale score was used to compare age-related differences. One OHI subject was removed from all the correlation analyses that involved the cognitive score, as her age was 86 years and no normative values for the cognitive test are available for individuals older than 85.

### Statistical analyses

All statistical analyses were conducted in SPSS version 24.0 (SPSS). Fisher’s z transformation was applied to all the correlation values calculated for the midbrain and MEG analyses before running statistical analyses. A split-plot analysis of variance (ANOVA) was performed to investigate effects of group (three levels: YNH, ONH, and OHI), effects of background condition (quiet and 4 noise levels: + 3 dB, 0 dB, -3 dB and -6 dB SNRs), effects of integration window (MEG only, 3 levels: 150, 300, and 500 ms), condition × group interactions, and window × group interactions on auditory temporal processing in both MEG and FFR data. The FFR dependent variables included RMS, quiet-to-noise correlation, and stimulus-to-response correlation, and the MEG dependent variable was reconstruction accuracy. Because of the significant age differences in the older groups, follow-up Analyses of Covariance (ANCOVAs) were performed with the two older groups for each analysis and covaried for age to determine if the age differences confounded the hearing loss effects. Condition × group interactions were assessed to determine if increasing levels of noise affect the younger and older groups differently. Follow-up ANOVAs were performed to test for group effects in the quiet or noise conditions separately when condition × group interactions were observed, and post-hoc t-tests were performed when main effects of group were observed. The Greenhouse-Geisser test was used when the Mauchly’s sphericity test was violated. The non-parametric Mann-Whitney U and Kruskal-Wallis H tests were used when Levene’s test of Equality of Variances was violated. Two-tailed Spearman’s rank correlation (ρ) was used to evaluate the relationships among cognitive score and midbrain and cortical parameters. The false discovery rate (FDR) procedure [89] was applied to control for multiple comparisons where appropriate.

## Results

### Speech intelligibility

The Kruskal-Wallis test showed significant differences in QuickSIN results among the 3 groups (χ^2^ = 27.566, *p* < 0.001). Post-hoc Mann-Whitney *U* tests showed that YNH (mean ± SD = -0.57 ± 1.13 dB SNR loss) performed significantly better than ONH (mean ± SD = 0.8 ± 1.25 dB SNR loss) (*p* = 0.002) and OHI (mean ± SD = 4.42 ± 3.23 dB SNR loss) (*p* < 0.001). OHI also performed significantly worse than ONH (*p* < 0.001). No significant correlations were found among QuickSIN and midbrain and cortical responses in both groups of older listeners (all p-values > 0.05 after correcting for multiple comparisons).

### Midbrain (EEG): Amplitude analysis

**Fig 2:**
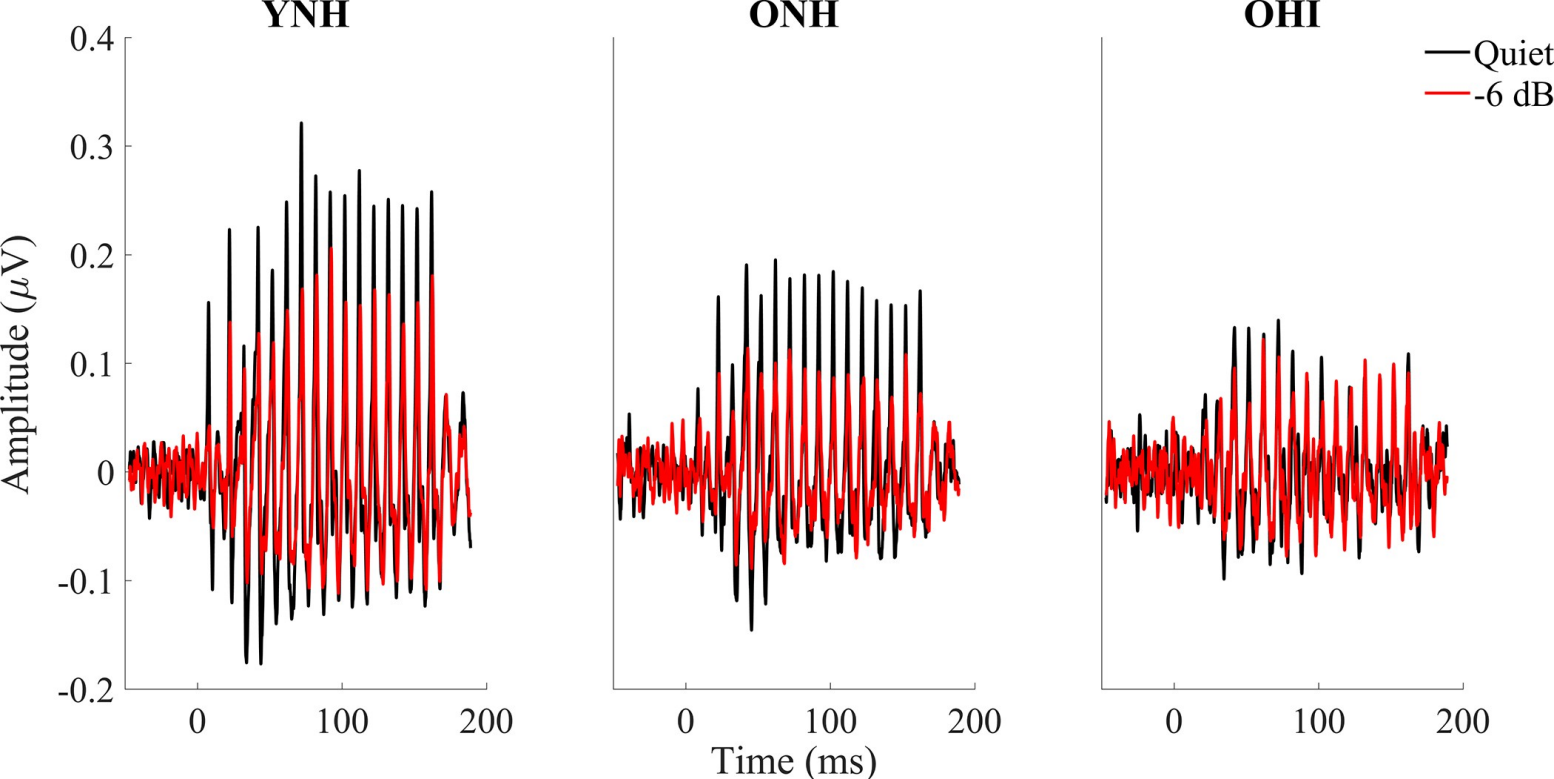
**Grand averages for YNH (n = 17, left column), for ONH (n = 15, middle column) and for OHI (n = 14, right column) of FFRs to the stimulus envelope recorded in quiet (black) vs -6 dB noise (red).** Overall results show a stronger response in younger adults in both the transition (18–68 ms) and steady-state (68–170 ms) regions. The presence or absence of hearing loss in older adults does not significantly affect the strength of the response.

**Fig 3:**
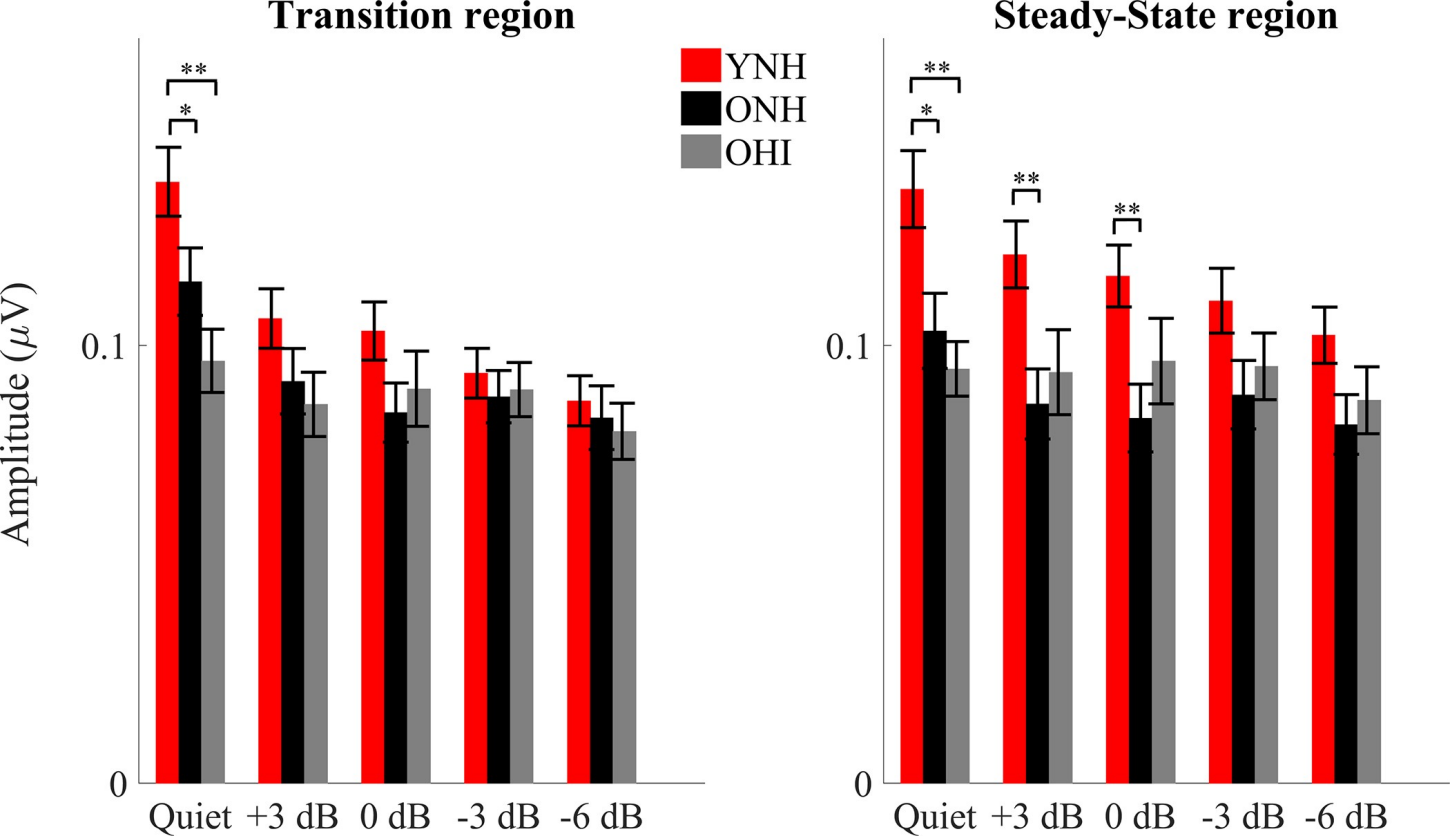
**RMS values ± 1 SE for ONH (black) and OHI (Gray) adults in the transition (*left*) and steady-state (*right*) regions for all of the noise levels tested.** Higher amplitudes were noted in the quiet condition in the YNH vs. either ONH or OHI. A steeper amplitude decline from quiet to noise conditions was noted in YNH compared to ONH or OHI groups. *p*-values in this figure refer to the results of the post-hoc independent t-tests analysis. *p < 0.05, **p < 0.01.

Fig 2 shows the grand average of FFRs to the stimulus envelope of YNH, ONH and OHI in quiet and the most severe noise condition (-6 dB SNR). The ability of midbrain neurons to synchronize in response to the stimulus was assessed by measuring the strength of the FFR via its RMS value. Overall results show a stronger response in younger adults in both the transition (18–68 ms) and steady-state (68–170 ms) regions than in either ONH or OHI participants. Older adults’ responses show evidence of degradation even in the quiet condition and are not much more degraded in the noise conditions. The presence of hearing loss in older adults does not significantly affect the strength of the response. Details follow below. Fig 3 displays the FFR RMS values for YNH, ONH and OHI for every SNR level tested.

### Transition region

A split-plot ANOVA including the quiet and four noise conditions showed a significant condition × group interaction (F_[2,43]_ = 5.486, *p* < 0.001), driven by a steeper amplitude decline from quiet to noise conditions in the YNH than in the ONH or OHI groups, as seen in Figs 2 and 3. A follow-up one-way ANOVA showed significant differences across the three groups in the quiet condition (F_[2,43]_ = 7.238, *p* = 0.002). Post-hoc t-tests showed larger amplitudes in YNH than in ONH (*p* = 0.048) and larger amplitudes in YNH than in OHI (*p* = 0.001), but no difference between ONH and OHI (*p* = 0.099). To determine effects of noise across SNR conditions, the split-plot ANOVA was performed for the noise conditions only and showed a main effect of condition, with decreasing SNRs resulting in decreased amplitudes across groups (F_[2,43]_ = 6.719, *p* = 0.001), but there were no main effects of group (F_[2,43]_ = 1.06, *p* = 0.355) and the condition × group interaction was not significant (F_[2,43]_ = 2.040, *p* = 0.079). A split-plot ANCOVA with age as covariate was performed across the five conditions with the two older groups. The age × condition interaction was not significant (F_[1,28]_ = 0.675, *p* = 0.616) and amplitude was not significantly different between the older groups (F_[1,28]_ = 0.703, *p* = 0.409).

### Steady-state region

Similar to the transition region, a split-plot ANOVA including the quiet and four noise conditions showed a significant condition × group interaction (F_[2,43]_ = 3.223, *p* = 0.003), driven by a steeper amplitude decline from quiet to noise conditions in the YNH than in the ONH or OHI groups (Figs 2 and 3). A follow-up one-way ANOVA showed significant differences among the three groups in quiet (F_[2,43]_ = 7.351, *p* = 0.002). Post-hoc t-tests showed larger amplitudes in YNH than in ONH (*p* = 0.014) and larger amplitudes in YNH than in OHI (*p* = 0.001), but no amplitude differences were observed between ONH and OHI (*p* = 0.426). The split-plot ANOVA for the noise conditions showed a main effect of noise, with decreasing SNRs resulting in decreased amplitudes across groups (F_[2,43]_ = 6.505, *p* = 0.001) and a main effect of group (F_[2,43]_ = 3.761, *p* = 0.031), but the noise level × group interaction was not significant (F_[2,43]_ = 1.906, *p* = 0.100). Follow-up MANOVA applied to RMS values from all the noise conditions tested revealed greater amplitudes in YNH than ONH (F_[1,31]_ = 7.409, *p* = 0.011) but there were no significant differences in amplitude between YNH and OHI (F_[1,30]_ = 3.197, *p* = 0.084) or between ONH and OHI (F_[1,28]_ = 0.566, *p* = 0.458). A split-plot ANCOVA with age as covariate was performed across the five conditions with the two older groups. The age × condition interaction was not significant (F_[1,28]_ = 2.736, *p* = 0.054) and the older groups were not significantly different (F_[1,28]_ = 0.175, *p* = 0.679).

### Midbrain (EEG): Quiet-to-noise correlation analysis

**Fig 4:**
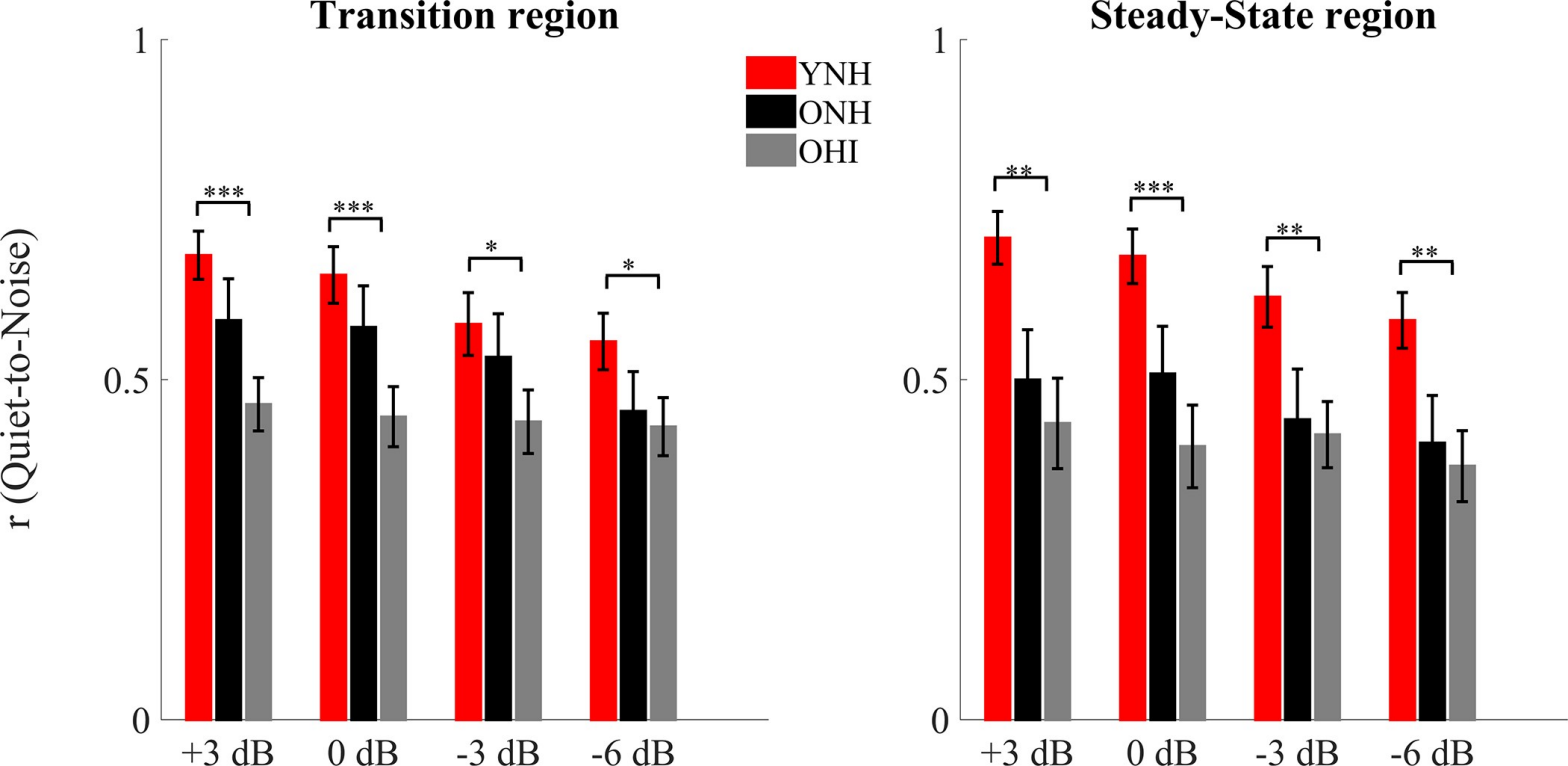
**Pearson correlation coefficients ± 1 SE of the quiet-to-noise correlation for YNH (red), ONH (black) and OHI (gray) in the transition (left) and steady-state (right) regions for all of the noise levels tested.** Significant differences were noted between YNH and OHI but not between YNH and ONH or ONH and OHI. *p*-values in this figure refer to the results of the post-hoc independent t-tests analysis. *P < 0.05, **p < 0.01, ***p < 0.001.

In order to analyze the robustness of the response profile in noise (the degradative effect of noise), a linear (Pearson) correlation was calculated between the average response (Fig 4) obtained in quiet and those obtained in noise, for both the transition and steady-state regions for each participant.

### Transition region

There was a significant condition × group interaction (F_[2,43]_ = 3.230, *p* = 0.005) that was driven by a steeper decline in correlation values with decreasing SNR in YNH vs. OHI (F_[1,30]_ = 3.661, *p* = 0.025) that was not seen between YNH and ONH (F_[1,32]_ = 1.344, *p* = 0.280) or between ONH and OHI (F_[1,28]_ = 2.535, *p* = 0.080). There was a main effect of condition, and correlations were lower with decreasing SNRs across groups (F_[2,43]_ = 13.787, *p* < 0.001). There was also a main effect of group across SNRs (F_[2,43]_ = 3.924, *p* = 0.027). Post-hoc t-tests showed significantly stronger quiet-to-noise correlations in YNH than in OHI at all the noise conditions tested (+3: *p* < 0.001, 0: *p* < 0.001, -3: *p* = 0.024, and -6: *p* = 0.031), but no differences between YNH and ONH (all *p*-values > 0.05) or between ONH and OHI (all *p*-values > 0.05). The Mann-Whitney test was used for +3, 0 and -6 dB SNR, because the Levene’s test of Equality of Variances was violated in those circumstances). A split-plot ANCOVA with age as covariate was performed across the four conditions with the two older groups. The age × condition interaction was not significant (F_[1,28]_ = 0.910, *p* = 0.451) and the older groups were not significantly different (F_[1,28]_ = 1.122, *p* = 0.299).

### Steady-state region

There was no significant condition × group interaction (F_[2,43]_ = 2.198, *p* = 0.059). There was a main effect of condition and correlations were lower with decreasing SNRs across groups (F_[2,43]_ = 14.69, *p* < 0.001). There was a main effect of group across SNRs (F_[2,43]_ = 5.207, *p* = 0.009). Post-hoc t-tests showed significantly larger correlations in YNH than in OHI (*p* = 0.012), but not between YNH and ONH (all *p*-values > 0.05). A split-plot ANCOVA with age as covariate was performed across the four conditions with the two older groups. The age × condition interaction was not significant (F_[1,28]_ = 0.212, *p* = 0.887) and the older groups were not significantly different (F_[1,28]_ = 0.044, *p* = 0.835).

### Midbrain (EEG): Stimulus-to-response correlation

The correlation between the clean stimulus and neural response was calculated in order to quantify the ability of the brain to follow the auditory input. No differences were observed among the groups in the quiet condition, but in noise, the YNH group had significantly higher stimulus-to-response correlations than the ONH or OHI groups. However, no significant age × condition interaction was found across the five conditions.

A split-plot ANOVA including the quiet and four noise conditions showed no main effect of condition (F_[2,43]_ = 1.482, *p* = 0.226). There was a main effect of group (F_[2,43]_ = 5.054, *p* = 0.011), but there was no significant condition × group interaction (F_[2,43]_ = 1.77, *p* = 0.086). Post-hoc t-tests showed that YNH had higher overall correlation values than ONH (*p* = 0.045) and OHI (*p* = 0.025), but there were no differences in overall correlation values between the OHI and ONH groups (*p* = 0.961). A split-plot ANCOVA with age as covariate was performed across the five conditions with the two older groups. The age × condition interaction was not significant (F_[1,28]_ = 1.022, *p* = 0.399) and the older groups were not significantly different (F_[1,28]_ = 0.836, *p* = 0.369).

### Cortex (MEG): Reconstruction of the attended speech envelope

**Fig 5:**
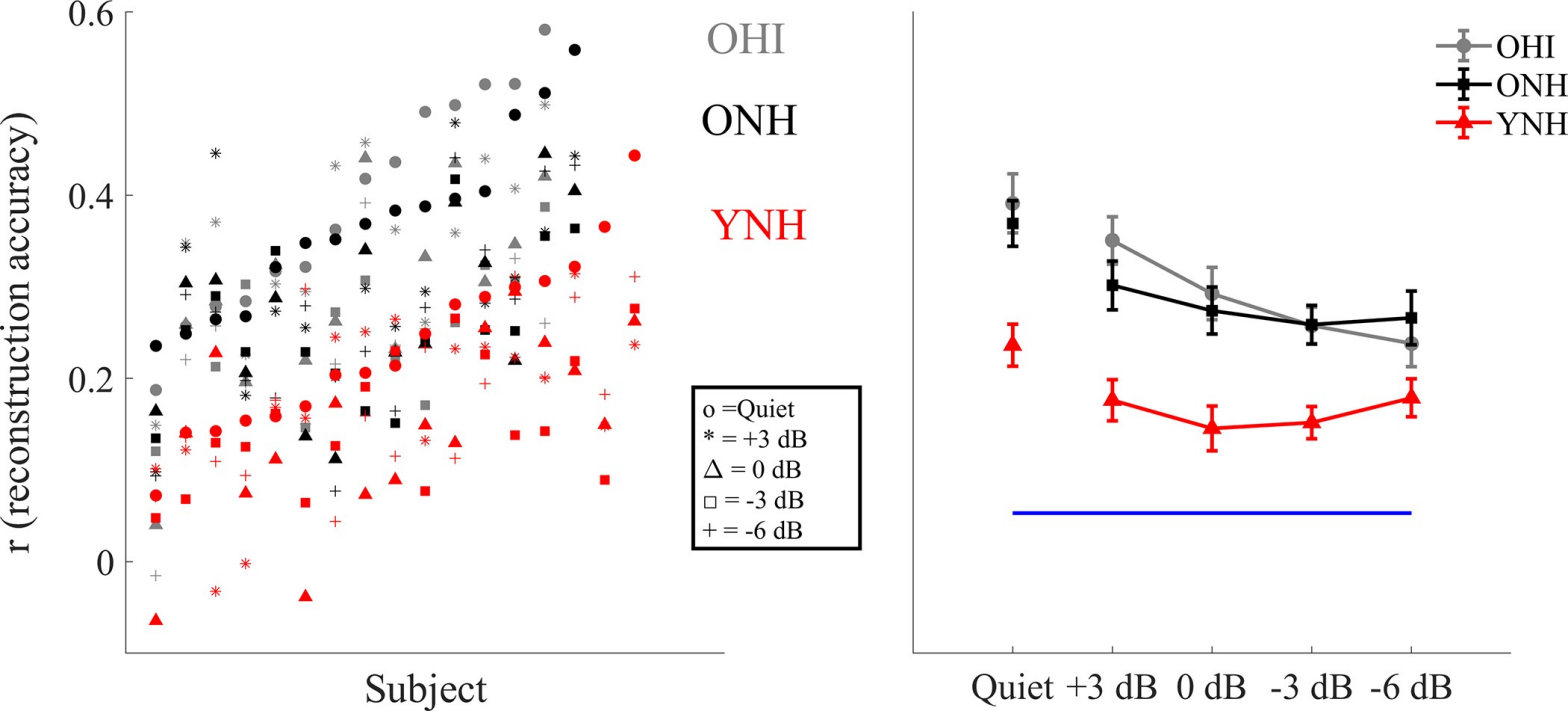
Correlation values measured in the MEG experiment. Left: plots of *r* values for each YNH (red), ONH (black) and OHI (gray) participant at each condition tested (o = Quiet, * = +3 dB, Δ = 0 dB, Υ = -3 dB, + = -6 dB) plotted in ascending noise order with respect to the quiet condition. Right: Reconstruction accuracy value 1 ± SE of the speech envelope of the foreground for YNH, ONH and OHI in quiet and in noise. The bottom horizontal line shows the noise floor. Older adults’ reconstruction fidelity is significantly better than that of the younger adults at all of the noise levels tested, but at -6 dB in OHI. No significant differences and no interactions were found between ONH and OHI.

The ability to reconstruct the low-frequency speech envelope from cortical activity is a measure of the fidelity of the neural representation of that speech envelope [19, 85]. Fig 5 shows the correlation values for each single individual tested at each of the SNRs condition tested, plotted in ascending order with respect to the quiet condition (left) and the grand average ± standard error of the reconstruction accuracy (right) for YNH, ONH and OHI for all the noise levels tested. All of the reconstruction values were significantly higher than the noise floor (all *p*-values < 0.01).

A split-plot ANOVA including the quiet and four noise conditions showed a main effect of condition (F_[2,43]_ = 22.699, *p* < 0.001) and a significant condition × group interaction (F_[2,43]_ = 2.434, *p* = 0.016). A follow-up one-way ANOVA showed significant differences across the three groups in the quiet condition (F_[2,43]_ = 9.926, *p* < 0.001). Post-hoc t-tests showed larger amplitudes in YNH than in ONH (*p* = 0.005) and larger amplitudes in YNH than in OHI (*p* = 0.001), but not between ONH and OHI (*p* = 0.817). To determine effects of noise across SNR conditions, the split-plot ANOVA was performed for the noise conditions and showed a condition × group interaction (F_[2,43]_ = 3.149, *p* = 0.006), driven by a significant decline in reconstruction values with decreasing SNR in the OHI (*p* = 0.005) that was not seen in YNH (*p* = 0.525) or ONH (*p* = 0.141). There was a main effect of condition across groups (F_[2,43]_ = 8.531, *p* < 0.001) and a main effect of group (F_[2,43]_ = 11.567, *p* < 0.001). Follow-up t-tests showed that the YNH group had lower reconstruction values than the ONH and the OHI groups at all SNRs (all p < 0.004), except at the -6 SNR condition at which the values between YNH and OHI were not significantly different (p = 0.265). There were no significant differences in reconstruction value between the older groups at any SNR (all p > 0.385). A split-plot ANCOVA with age as covariate was performed across the five conditions with the two older groups. The age × condition interaction was not significant (F_[1,28]_ = 0.211, *p* = 0.932) and the older groups were not significantly different (F_[1,28]_ = 0.308, *p* = 0.584).

### Effect of the integration window

**Fig 6:**
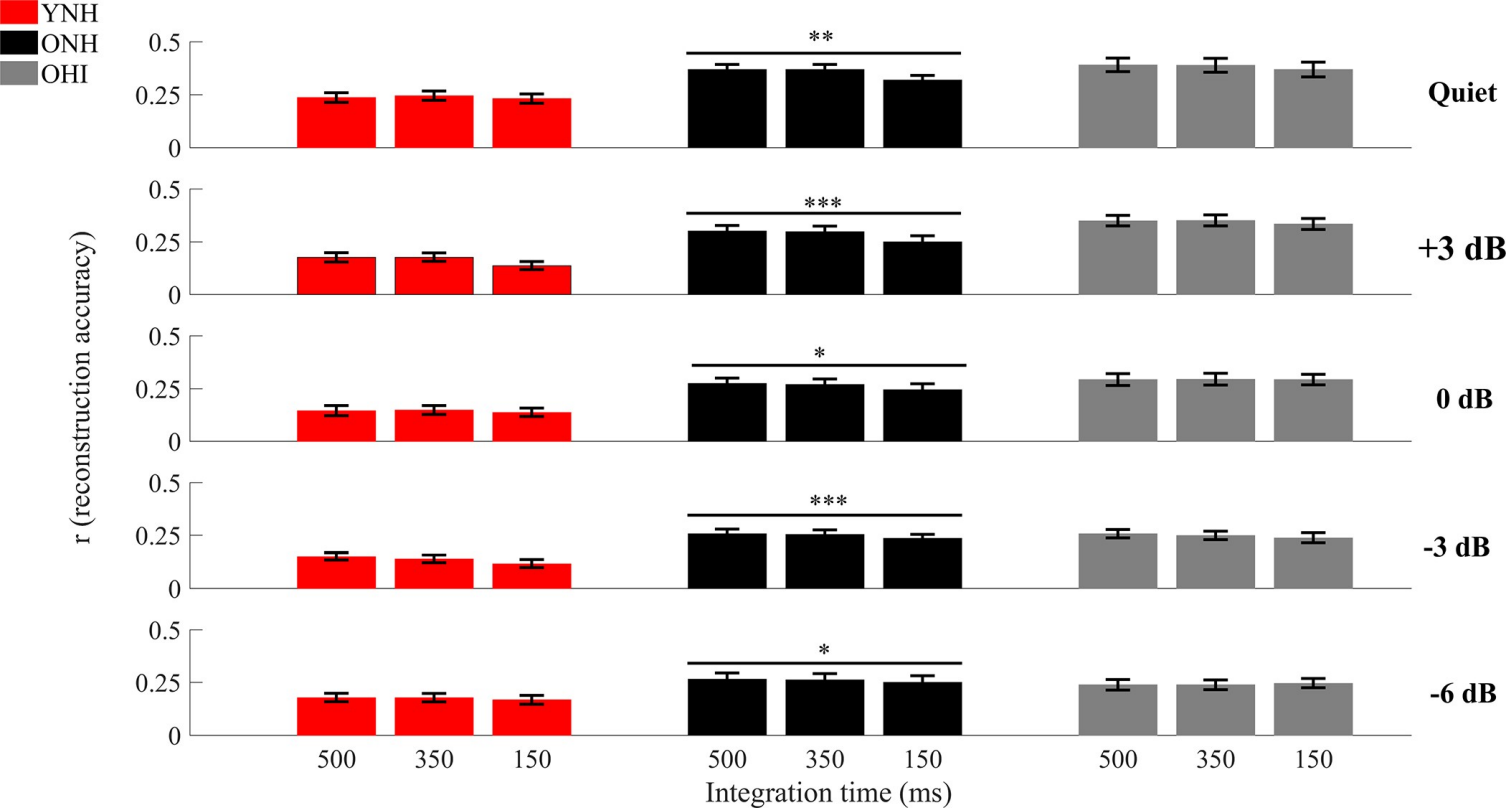
Reconstruction accuracy in quiet and at all the noise conditions tested for the 3 integration windows tested: 500, 350, and 150 ms. Significant differences across the 3 integration windows were found only in ONH in both quiet and at all the noise conditions tested. The size of the integration window seems to be playing a critical role only in older adults with normal hearing. *p < 0.05, **p *<* 0.01, ***p < 0.001.

The fidelity of the reconstruction was also tested at different integration windows and the correspondent correlation values are shown in Fig 6, with a focus on the statistical measures pertinent to the integration window. A split-plot ANOVA, including the quiet and four noise conditions, were performed to test for group differences in the decline of reconstruction value with smaller integration windows and showed a significant window × group interaction (F_[2,43]_ = 4.718, *p* = 0.010) and a significant window × condition interaction (F_[2,43]_ = 5.301, *p* < 0.001). After correcting the *p*-values for multiple comparisons, results from follow-up split-plot ANOVA showed significant differences between integration windows only in ONH (F_[2,28]_ = 14.954, *p* = 0.001, F_[2,28]_ = 20.457, *p* < 0.001, F_[2,28]_ = 5.185, *p* = 0.034, F_[2,28]_ = 16.094, *p* < 0.001, F_[2,28]_ = 5.048, *p* = 0.037 in quiet, +3, 0, -3 and -6 dB respectively), while no significant differences were found in YNH and OHI (all p-values > 0.05). A split-plot ANCOVA with age as covariate was performed across the five conditions and three integration windows with the two older groups. The age × condition interaction was not significant (F_[1,28]_ = 0.161, *p* = 0.956), the age × window interaction was not significant (F_[1,28]_ = 0.097, *p* = 0.908), and the groups were not significantly different (F_[1,28]_ = 0.596, *p* = 0.447).

### Reconstruction of the unattended speech envelope

A split-plot ANOVA for the four background noise conditions showed a main effect of condition (F_[2,43]_ = 4.089, *p* = 0.008), a main effect of group (F_[2,43]_ = 7.269, *p* = 0.002), but no condition × group interaction (F_[2,43]_ = 2.078, *p* = 0.060). Post-hoc tests revealed lower reconstruction values in the YNH group compared to ONH (p = 0.009) and OHI (p = 0.009), but no differences between ONH and OHI (p = 0.998). A split-plot ANCOVA with age as covariate was performed across the five conditions with the two older groups. The condition × group interaction was not significant (F_[1,28]_ = 0.750, *p* = 0.533) and the older groups were not significantly different (F_[1,28]_ = 0.186, *p* = 0.670).

### Relationships among cognitive, midbrain and cortical data

**Fig 7:**
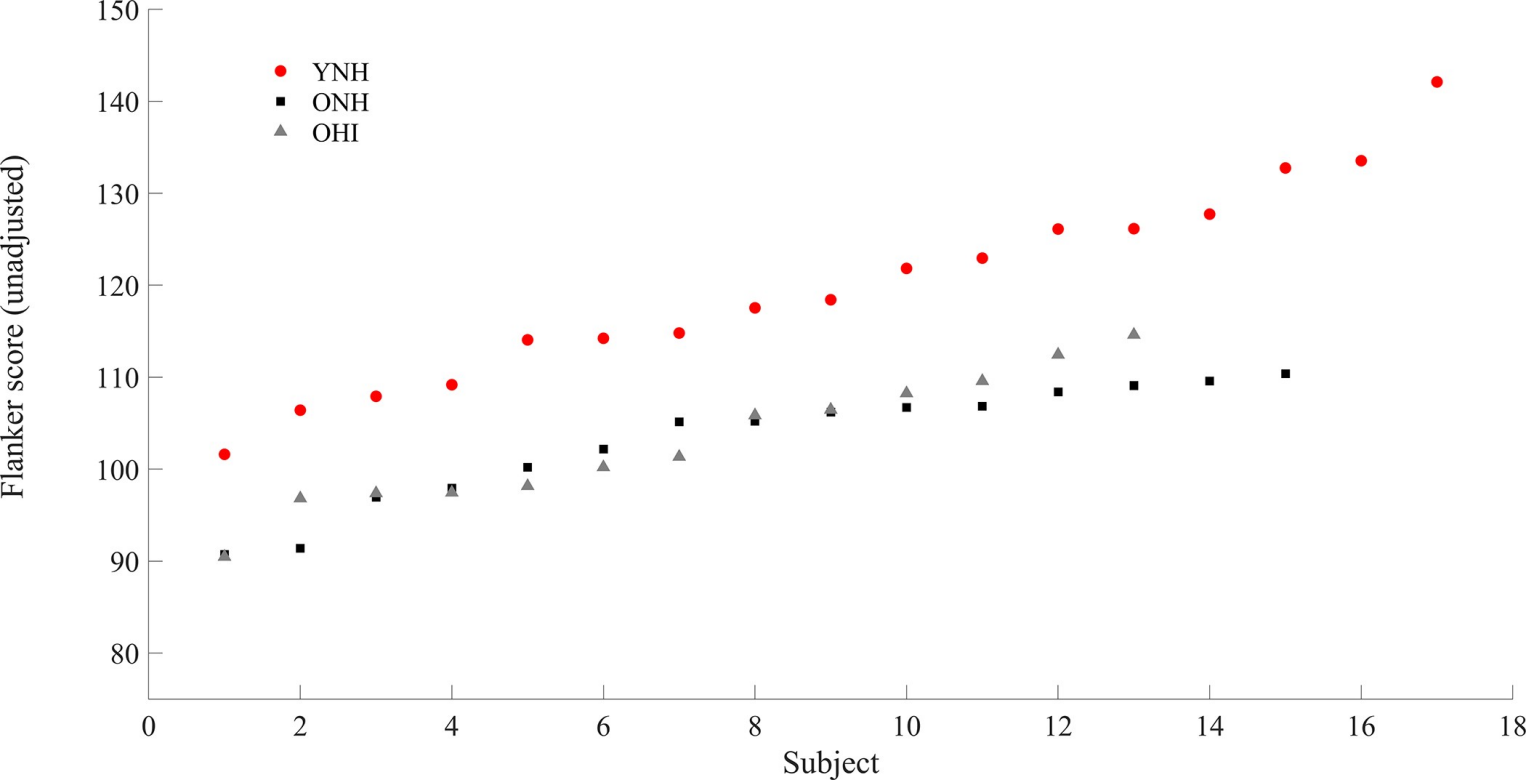
Unadjusted scale score of the Flanker Inhibitory Control and Attention test for each subject plotted in ascending order. NHY scored significantly better than ONH and OHI, but no significant differences were found between ONH and OHI. ***p < 0.001.

Fig 7 shows the results of the Flanker Inhibitory Control and Attention test for each subject. The Flanker Inhibitory Control and Attention test showed significant differences among the three groups (F_[__1__,__42__]_ = 20.516, *p* < 0.001). Follow-up t-tests showed that YNH had significantly higher scores than ONH (t_[__30__]_ = 5.232, *p* < 0.001) and OHI (t_[__28__]_ = 4.859, *p* < 0.001), while no significant differences were found between ONH and OHI (t_[__26__]_ = 0.05, *p* = 0.961). The Flanker score was evaluated with respect the brain measures: RMS values, quiet-to-noise correlations and stimulus-to-response correlations for EEG, and correlation values of the attended speech envelope at the integration window of 500 ms for MEG. The choice of this integration window was dictated by the desire to compare behavioral scores with the neural response that represented the best encoding of the speech envelope for both older and younger adults. Significant negative correlations (lower score associated with higher reconstruction accuracy) were found between the Flanker Inhibitory Control and Attention test score and the cortical response (average cortical decoding accuracy across all the noise levels) (ρ = -0.621, p = 0.013) in ONH, but not in YNH (ρ = 0.431, p = 0.084) or in OHI (ρ = 0.429, *p* = 0.144). No significant correlations were found among the Flanker Inhibitory Control and Attention test score and midbrain responses (average quiet-to-noise correlation across all the noise levels in the steady-state region and average stimulus-to-response correlation across all the noise levels) in any the three groups tested (all *p* > 0.05). Similarly, no significant correlations were found among midbrain and cortical responses in YNH and ONH (*p* > 0.05). However, significant positive correlations were found in OHI between cortex and average midbrain quiet-to-noise correlations across all the noise levels in the steady-state region (ρ = 0.719, *p* = 0.004), but not between cortex and average midbrain stimulus-to-response correlation across all the noise levels (ρ = 0.336, *p* = 0.240).

## Discussion

Although hearing loss affects speech understanding in noise, no significant differences in encoding of speech signals in noise between ONH and OHI were seen in either the midbrain or the cortex, with the exception of the integration window analysis, where differences were found between the two older listener groups. Furthermore, two additional results that did not meet our expectations were found. The first one was the absence of a significant negative correlation between cognitive performance and cortical responses in OHI, while the second one was the significant correlation between cortex and midbrain observed in OHI. These results suggest that peripheral hearing loss might alter the relationship between the two areas of the auditory system investigated.

### Midbrain (EEG): Amplitude analysis

Significant differences were found between YNH and the two groups of older listeners in quiet, but no significant differences between ONH and OHI. These findings are consistent with a previous study [17], in which FFRs were recorded in response to the speech syllable /u/ (in quiet) in normal-hearing and hearing-impaired participants. Those results showed that, despite a higher degree of degradation of the F0 of the envelope response in subjects with peripheral hearing loss, the two groups did not differ significantly.

In another study conducted in older adults [44], the authors found a higher representation of the envelope in participants with hearing loss. Interestingly, these differences were exacerbated by the presence of background noise. The differing results between the current study and that one might be explained by the design dissimilarities between the two studies. First, the stimuli used in the studies differed not only in duration, but in the composition of the spectral components. The current study used a 170 ms /da/, with a relatively low amplitude noise burst during the first 10 ms and a fundamental frequency of 100 Hz, which allowed analysis of both the transient and the steady-state response. Conversely, the stimulus used by other colleagues [44] was very short (40 ms), limiting the analysis to only the transition region, had a fundamental frequency that linearly rose from 103 to 125 Hz, and had a shorter noise burst (5 ms) that was higher in amplitude than the one used here. The higher-amplitude noise burst of the 40-ms /da/ may activate a larger population of neurons, and because the OHI would have broader tuning bandwidths [90], the differences between ONH and OHI might be more pronounced. The group differences in the study [44] were more apparent for the amplified /da/ stimulus than the unamplified stimulus, and when the /da/ was presented in noise rather than in quiet. The noise stimulus in that study was pink noise, and again, the OHI participants may have higher response amplitudes to pink noise than would be expected to a single talker.

It is also possible that the groups in the two different studies differed in the type of cochlear pathology that dominated the sensorineural hearing loss in the individual listeners. Individuals with sensorineural hearing loss may be affected by different degrees of cochlear gain loss vs. cochlear synaptopathy (as modeled by other colleagues[91]), and individuals with cochlear gain loss may have exaggerated temporal envelopes. The individuals in previous study [44] may have been affected by cochlear gain loss to a greater extent than cochlear synaptopathy compared to the individuals in the current study.

If it is assumed that peripheral hearing loss may somehow play a role in the disruption of the balance between subcortical inhibitory and excitatory mechanisms [11–16], it is possible that the lack of significant differences in response amplitude between ONH and OHI could be explained by poorer ONH hearing thresholds in the current study compared to those reported by other colleagues [44]in ONH group. In fact, the envelope response in the ONH group of the current study may be somewhat exaggerated due to PTA thresholds that are towards the limit of the normal hearing range, though this scenario seems unlikely based on these findings.

The results might also appear to contradict previous findings that have demonstrated exaggerated amplitudes associated with hearing loss in animal models [41, 92]. However, two important differences with respect to this experiment may limit the comparisons with these animal models. The first one is the etiology of hearing loss. Sensorineural hearing loss in chinchillas was induced by noise overexposure, while these subjects did not report any history of unusually high noise exposure. Second, the increase in response amplitude reported by other colleagues [41] was observed only in fibers whose characteristic frequency was between 1 and 2 kHz. The FFR here had a fundamental frequency of 100 Hz, a frequency at which results from a previous study [41] did not find any increase in amplitude response. However, our approach to average sweeps with alternating polarity to minimize stimulus artifact and cochlear microphonic significantly minimize the contribution of high frequencies (e.g. formants), thus we cannot rule out that the lack of exaggerated response in our data might be due to a considerable reduction of amplitude in the formants.

### Midbrain (EEG): Robustness of the envelope to noise

The stimulus-to-response correlation analysis showed no significant differences between ONH and OHI, consistent with what was observed in the amplitude analysis. Conversely, the quiet-to-noise correlation, which was used to investigate the effect of noise on the latency of the response, did reveal differences between ONH and OHI.

### Quiet-to-noise correlation analysis

YNH showed a significantly more robust response in noise than OHI in both the transition and steady-state regions, but no significant differences were found between YNH and ONH, or between ONH and OHI. Interestingly, in this analysis significant declines in the robustness of the response across noise conditions in the transition and steady-state regions were observed in YNH and ONH, but not in OHI, suggesting that peripheral hearing loss further deteriorates the latency of the encoded stimulus envelope already affected by aging [9, 10, 18, 19, 32, 93], A prolongation in timing will affect the strength of the correlation between responses obtained in quiet and noise. Therefore, in the OHI group, even in relatively favorable conditions (e.g. +3 dB), the timing of the response is already so significantly compromised that the response is close to the floor, and further increases in noise do not increase the degradation in the response in OHI.

These results might be informed by those reported by other colleagues [94] who recorded auditory brainstem responses (ABRs) to click stimuli presented in quiet and in several levels of broadband noise. In young normal-hearing adults, the ABR Wave V latency is expected to increase with decreasing SNRs due to neural desynchronization. However, the slope of this latency increase was shallower in some of the study participants who had lower Wave I amplitudes and poorer performance on a temporal processing task. The authors [94] surmised that the ABR latency in noise may be a marker of cochlear synaptopathy. It is interesting that the decrease in correlation values was found in the ONH group but not in the OHI group. If the ONH group was significantly affected by peripheral hearing loss, synaptopathy, or loss of auditory nerve fibers, one might expect to find a group × noise level interaction between ONH and YNH, but both groups experienced a similar loss of correlation value with increased noise levels.

### Stimulus-to-response correlation

The YNH group had significantly higher stimulus-to-response correlations than either ONH or OHI groups, but there were no differences between ONH and OHI groups. These results contrast with the quiet-to-noise correlations that showed an overall effect of hearing loss but not of aging. The differences between the analyses may explain these seemingly conflicting results. The quiet-to-noise correlation is affected by the timing of the response, which is compromised by reduced audibility [38]. The stimulus-to-response correlation is affected by the degradation of the neural stimulus envelope, and the analysis is independent of the latency delay. The fact that YNH had a better representation of the stimulus with respect to ONH and OHI and that no differences were found between the two older adult groups speaks in favor of an age-related degradation of the response, rather than a degradation associated with hearing loss.

### Cortex (MEG): Reconstruction of the speech envelope

The results from the cortical analysis confirm the existence of overrepresentation of the response envelope in older adults with and without peripheral hearing loss. The reconstruction accuracy of OHI is significantly higher than YNH in both quiet and noise, a finding that is consistent with other studies showing an exaggerated cortical response associated with age [18–22, 95]. In our previous experiments [18, 19], we speculated that this abnormally high cortical response could be due to a mix between an age-related imbalance between inhibitory and excitatory mechanisms and age-related cognitive deficits. However, it could not be ruled out that loss of hearing sensitivity could be the driving factor. The current study suggests that indeed age may be the driving factor behind changes at the cortical level. Few significant differences in cortical measures between ONH and OHI were observed in any of the conditions tested. Had peripheral hearing loss been the driving factor in explaining this overrepresentation, significantly higher reconstruction accuracy in OHI with respect to ONH would have been expected. These findings were also confirmed in the analysis of the unattended speech, which was significantly higher in the two older listener groups with respect to the younger participants.

It is important to point out that a recent study of hearing-impaired older adults [45] showed a significant decrease in the attentional modulation of neural tracking with respect to normal-hearing older adults. A plausible explanation for the disagreement between the current findings and those ones reported in the above mentioned hearing-impaired study [45] may be linked to the fact that participants in that study wore hearing aids programmed with an amplification scheme that incorporated slow-acting compression. Compression algorithms, even with slow release times, have been shown to distort to some degree the speech envelope [96], thus leading to possible degradation of the neural response, and this degradation may have affected the response.

Interestingly, this same study [45] also showed that listeners with poorer hearing had less change in neural tracking from the most to least favorable SNRs tested, whereas our study found that neural tracking of speech envelope declined with decreasing SNR in OHI group, but not in ONH or YNH groups. We speculate that this discrepancy could also be linked to the compression algorithm used in [45], which could have distorted the speech envelope.

The only difference in speech envelope reconstruction between ONH and OHI was observed in the integration windows analysis. In our previous study [19], it was shown that narrowing the integration window negatively affected the reconstruction accuracy of ONH, but had no significant effect on YNH. We then speculated that this result was consistent with previous psychoacoustic [4, 8] and electrophysiological [21, 97] experiments showing that older adults had more problems adapting to changes in temporal parameters, and therefore expected to see similar results in OHI. The difference may arise due to mechanisms having been altered by hearing loss; several studies have shown that loss of auditory sensitivity may affect the reorganization of different areas of the brain, thus leading to different speech encoding strategies in ONH and OHI [46–49]. It is therefore possible that the late latency response might have been altered to the point that increasing the reconstruction accuracy to 500 ms would not lead any additional benefit to the encoding of the stimulus envelope. More studies will be required to elucidate this unexpected result.

### Relationships among neural responses and cognitive function

One of the most important and intriguing results of our previous experiment [18] was the lack of correlation between midbrain and cortex and a significantly negative correlation between cortex and cognitive scores in ONH. These findings contrasted the findings in the current study of a significant correlation between cortical and midbrain responses, and a lack of correlation between cortex and cognitive scores in the OHI group. In the previous study, we speculated that the lack of correlation between midbrain and cortex was consistent with results from a recent animal study [34] that showed the existence of compensatory central gain increases that may restore the representation of the auditory object in cortex even when the input from the brainstem is severely degraded. The negative correlation between cortex and cognitive scores in the ONH group supported the hypothesis in the previous study that an overrepresentation in the cortex was not a biomarker that represents an advantageous response of the brain but rather, an abnormally high neural activity that could indicate failure in processing auditory information, as also reported by a recent study [43].

The presence of peripheral hearing loss was therefore a critical factor for this analysis, because several studies have shown how loss of auditory sensitivity affects the reorganization of different areas of the brain [46–49]. The results indeed show a different relationship between midbrain and cortex in OHI, as indicated by a significant positive correlation between cortical and midbrain responses. This finding may suggest that peripheral hearing loss is associated with higher interdependence between midbrain and cortex, consistent with recent findings [31]. Specifically, the correlation reported by other colleagues [31] was seen only in the older subjects, many of whom had significant hearing loss, and it was not seen in the younger subjects.

Although the ONH group showed a negative correlation between the Flanker Inhibitory Control and Attention test score and the reconstruction value of the cortical envelope, neither the YNH nor the OHI groups showed a signification relationship between the two measures; in fact, the direction of the correlation for both YNH and OHI groups was in a positive rather than in a negative direction. This positive relationship was perhaps not surprising in younger adults. In fact, in a healthy and fully functioning auditory brain with the proper balance of inhibitory and excitatory neurotransmission and preserved connectivity between different cortical regions, one might expect that the neural response would follow cognitive function. In other words, the better the reconstruction accuracy of the target speech envelope, the better the performance in an attention and inhibitory task. In the OHI group, however, this was an unexpected finding given the hypothesis that overrepresentation of the cortical response does not indicate enhanced temporal processing. We previously noted a midbrain-cortical association in the OHI that was not seen in the ONH, suggesting a tighter link between subcortical and cortical regions in individuals with hearing loss [31]. Similarly, in cortex, hearing loss leads to reorganization, with a loss of gray matter volume in primary auditory cortex [48] and higher activation of frontal cortical areas [46, 47, 98]. We speculate that hearing loss affects cognitive-cortical relationships differently than the presence of aging alone. This cognitive-cortical relationship may be elucidated in a future study that combines MEG and pupillometry to provide an online measure of cognitive effort during the speech perception tasks.

### Speech in noise

As expected, the scores of the speech-in-noise test showed that YNH performed significantly better than both groups of older listeners. Interestingly, despite significant differences in PTA and speech-in-noise score between ONH and OHI, an overall lack of significant differences between these two groups were found in the neural response. Furthermore, no significant correlations were found among the QuickSIN, midbrain, and cortical responses in either group of older listeners. We argue that two plausible explanations for this disagreement may exist. The first is linked to the different type of background noise utilized. The background noise in the QuickSIN was represented by a four-talker babble, which provides more energetic masking, while for the EEG and MEG recordings it was represented by a single talker, which provides more informational masking.

The second explanation is related to the fact that QuickSIN relies on short sentences of ~7 words in length while these neural recordings were obtained with participants listening to 1 minute of speech material. Factors such as cognitive load, inhibitory processes and type of context of the story may play a larger role for an entire minute of speech than in a short sentence. The use of QuickSIN was motivated by its wide use in clinical settings. However, the results of this experiment would suggest that a different speech-in-noise test bearing closer similarities to this speech material should probably be adopted in future experiments.

### Limitations of the study

The main limitation of this study was the inability to recruit a larger number of aged-matched older adults with and without hearing loss, which would have allowed us to increase our statistical power. At issue is the difficulty of finding participants *across groups but at* similar ages despite the known association between age and hearing loss, compounded by the overall difficulty in finding participants that qualify for MEG. It is therefore not surprising that the age ranges of all ONH and all OHI participants were significantly different. The age-match problem was addressed by adding age as a covariate to our statistical analysis. While the results obtained are robust under the statistical analysis employed, given the limitations just described, we cannot rule out the possibility that the disagreement between some of these results with those reported by other colleagues [45] might be partially due to different statistical power between the two experiments.

## Conclusions

The overall results of this study bring additional support to the hypothesis that central temporal auditory deficits are critical factors in the communications problems experienced by older adults. Overall, subcortical and cortical responses each show no significant differences between older normal-hearing and older hearing-impaired listeners, suggesting that aging is a driving factor in explaining degradation of speech comprehension in noise. What remains to be elucidated from this experiment is the significant correlation between cortical and midbrain responses in the participants with hearing loss and the lack of sensitivity to changes in the integration window in OHI. At this point, we can only speculate that this relationship is due to functional changes in the brain caused by the presence of hearing loss. Future directions will focus on using the biomarkers identified in this study to assess the efficacy of auditory training techniques. Specifically, it will be critical to understand if an improvement in speech understanding is associated with changes in the neural response, such as reduced degradative effects of noise on midbrain responses and reduced overrepresentation of the cortical response.
